# Small for gestational age and risk of childhood mortality: A Swedish population study

**DOI:** 10.1371/journal.pmed.1002717

**Published:** 2018-12-18

**Authors:** Jonas F. Ludvigsson, Donghao Lu, Lennart Hammarström, Sven Cnattingius, Fang Fang

**Affiliations:** 1 Department of Medical Epidemiology and Biostatistics, Karolinska Institutet, Stockholm, Sweden; 2 Department of Pediatrics, Örebro University Hospital, Örebro, Sweden; 3 Division of Epidemiology and Public Health, School of Medicine, University of Nottingham, Nottingham, United Kingdom; 4 Department of Medicine, Columbia University College of Physicians and Surgeons, New York, New York, United States of America; 5 Centre of Public Health Sciences, Faculty of Medicine, University of Iceland, Reykjavik, Iceland; 6 Department of Laboratory Medicine, Clinical Epidemiology Unit, Karolinska Institutet, Stockholm, Sweden; 7 Department of Medicine, Solna, Karolinska Institutet, Stockholm, Sweden; Cambridge University, UNITED KINGDOM

## Abstract

**Background:**

Small for gestational age (SGA) has been associated with increased risks of stillbirth and neonatal mortality, but data on long-term childhood mortality are scarce. Maternal antenatal care, including globally reducing the risk of SGA birth, may be key to achieving the Millennium Development Goal of reducing under-5 mortality. We therefore aimed to examine the association between SGA and mortality from 28 days to <18 years using a population-based and a sibling control design.

**Methods and findings:**

In a Swedish population study, we identified 3,795,603 non-malformed singleton live births and 2,781,464 full siblings born from January 1, 1973, to December 31, 2012. We examined the associations of severe (<3rd percentile) and moderate (3rd to <10th percentile) SGA with risks of death from 28 days to <18 years after birth. Children born SGA were first compared to non-SGA children from the population, and then to non-SGA siblings. The sibling-based analysis, by design, features a better control for unmeasured factors that are shared between siblings (e.g., socioeconomic status, lifestyle, and genetic factors). Hazard ratios (HRs) were calculated using Cox proportional hazards and flexible parametric survival models. During follow-up (1973–2013), there were 10,838 deaths in the population-based analysis and 1,572 deaths in sibling pairs with discordant SGA and mortality status. The crude mortality rate per 10,000 person-years was 5.32 in children born with severe SGA, 2.76 in children born with moderate SGA, and 1.93 in non-SGA children. Compared with non-SGA children, children born with severe SGA had an increased risk of death in both the population-based (HR = 2.58, 95% CI = 2.38–2.80) and sibling-based (HR = 2.61, 95% CI = 2.19–3.10) analyses. Similar but weaker associations were found for moderate SGA in the population-based (HR = 1.37, 95% CI = 1.28–1.47) and sibling-based (HR = 1.38, 95% CI = 1.22–1.56) analyses. The excess risk was most pronounced between 28 days and <1 year of age but remained throughout childhood. The greatest risk increase associated with severe SGA was noted for deaths due to infection and neurologic disease. Although we have, to our knowledge, the largest study sample so far addressing the research question, some subgroup analyses, especially the analysis of cause-specific mortality, had limited statistical power using the sibling-based approach.

**Conclusions:**

We found that SGA, especially severe SGA, was associated with an increased risk of childhood death beyond the neonatal period, with the highest risk estimates for death from infection and neurologic disease. The similar results obtained between the population- and sibling-based analyses argue against strong confounding by factors shared within families.

## Abstract 2

Leveraging a sibling-controlled design, Jonas Ludvigsson and colleagues examine whether SGA is associated with an increased risk of childhood death beyond the neonatal period in Sweden, and describe the most common causes of death in affected children.

## Author summary

**Why was this study done?:**

- Small for gestational age (SGA) occurs in more than 30 million infants every year.
- SGA has been associated with increased risks of stillbirth and neonatal mortality, but data on long-term childhood mortality are scarce.

**What did the researchers do and find?:**

- We evaluated the association between SGA and mortality from 28 days after birth to 18 years of age, using both general population and sibling comparators in more than 3.7 million Swedish children over a period of 40 years.
- Compared with non-SGA children, children born with severe SGA had an increased risk of death throughout childhood in both the population- and sibling-based analyses.
- The greatest risk increase associated with severe SGA was noted for deaths due to infection and neurologic disease.
- Similar but weaker associations were found for moderate SGA.

**What do these findings mean?:**

- Our findings provide insights on associations between SGA and causes of death in childhood. SGA cannot be reversed, and hence primary preventive strategies are needed to decrease the long-term risks of intrauterine growth restriction.
- Even if absolute risks are low, an opportunity to reduce the harms of SGA may exist in infectious disease prevention and treatment.

## Introduction

The Barker hypothesis proposes that intrauterine growth restriction may cause cardiovascular disease in middle and old age [1–3]. Some data indicate that fetal growth may also influence other diseases in adulthood [4,5], although confounding remains an issue [6].

Small for gestational age (SGA) refers to newborns with a low birth weight for gestational age, according to the reference curve for normal fetal growth or birth weight for gestational age. SGA, defined as either less than the 3rd percentile or less than the 10th percentile, has been associated with stillbirth and neonatal [7,8] and postneonatal mortality (death at 0–27 and 28–364 days of life, respectively) [8,9]. Despite the fact that SGA yearly occurs in more than 30 million infants worldwide [10], there are limited data on the association of SGA with mortality beyond the perinatal period. If such an association is confirmed, it may provide additional motivation for achieving the Millennium Development Goal of lowering under-5 mortality [11] and even mortality throughout childhood by globally reducing SGA births.

We are aware of 5 studies that examine the relationship between SGA and mortality beyond the first year of age. Two studies followed up children to the age of 5 years but not after that [12,13]. They found increased mortality, but did not report cause-specific mortality. Another study found that poor fetal growth (per quartile) was associated with an increased risk of death from cardiovascular disease, but did not look at overall mortality [14]. A fourth study reported cumulative mortality up to 14 years of age in children born SGA but did not calculate relative risks [15]. Finally, a Danish study reported an increased risk of death in children born SGA [16]. However, that study included children with malformations, which may substantially contribute to SGA-related mortality, and did not discriminate between severe (<3rd percentile) and overall (<10th percentile) SGA. Recent data have suggested that using a cutoff at the 3rd percentile (severe SGA), rather than the 10th percentile, may better reflect poor fetal growth [17]. Finally, a study using within-sibling comparisons—which, by design, feature a better control for factors that are unmeasured but potentially shared between siblings (e.g., socioeconomic status, lifestyle, and genetic factors)—is warranted.

In this study we evaluated the association between SGA and mortality from 28 days after birth to <18 years, using both general population and sibling comparators in more than 3.7 million Swedish children over a period of 40 years. We hypothesized that severe SGA (<3rd percentile), and to a smaller degree moderate SGA (3rd to <10th percentile), would be associated with increased mortality throughout childhood. We also hypothesized that such associations could not be entirely explained by genetic and early life risk factors.

## Methods

### Registers

Swedish registers include information on a personal identity number (PIN), uniquely assigned to all Swedish residents [18]. We used the PIN to link data on pregnancy outcomes in the Swedish Medical Birth Register (MBR) [19] and deaths in the Swedish Cause of Death Register [20]. The MBR started in 1973, and includes information on pregnant women and their offspring. The register covers more than 98% of all births in Sweden. The data collection starts at the first antenatal visit (around 12 gestational weeks). Since 1982, all data have been registered using standardized charts. The Cause of Death Register has recorded the time and cause of all deaths in Sweden since 1952. Causes of death are coded in accordance with the International Classification of Diseases (ICD) system.

Information on the highest level of maternal education was obtained from the yearly updated Swedish Register of Education. Data were also obtained from the Swedish National Patient Register [21], a register that includes information on inpatient discharge records from 1964 onward (nationwide since 1987) and hospital-based outpatient specialist care from 2001 onward. The positive predictive value for most diagnoses in the National Patient Register is 90% [21]. Follow-up was determined through the Total Population Register, which records data on family relationships and life events, including birth, death, name change, marriage and divorce, and migration to and from Sweden [22]. A portion of this register goes under the name of the Multi-Generation Register [23], which allows the identification of parents for all live births included in the present analyses.

### Participants

In Sweden, between January 1, 1973, and December 31, 2012, there were 3,997,744 singleton live births recorded in the MBR. We excluded 10,889 neonatal deaths (death of a live birth from 0 to 27 days of life), as we intended to study mortality from 28 days after birth and onwards as the primary outcome. We further excluded births with missing or invalid PINs (*n* = 20,003) and births with missing information on gestational age (*n* = 8,977), maternal age (*n* = 4,376), date of birth (*n* = 219), or infant sex (*n* = 2), or missing or implausible values of birth weight (*n* = 15,086). Finally, to exclude the contribution of congenital malformations to the association between SGA and mortality, we excluded 124,589 births diagnosed with major malformations during the first year of life. Information on malformations was ascertained through the MBR and the Swedish National Patient Register. The specific ICD codes (ICD-8 during 1973–1986, ICD-9 during 1987–1996, and ICD-10 during 1997–2012) for malformations are provided in S1 Table. As a result, we constructed a population cohort of 3,795,603 singleton live births surviving the neonatal period, and followed the cohort to death, emigration, the day before the 18th birthday, or December 31_,_ 2013, whichever came first.

An association between SGA and childhood mortality might be attributable to unmeasured genetic or non-genetic confounders. Using information on mothers (identified from the MBR) and fathers (identified from the Multi-Generation Register), we constructed a cohort of 2,781,464 full siblings (73% of the population cohort). Within this cohort, we used a sibling control design in which children born SGA were compared with their non-SGA full siblings. The purpose of this design is to eliminate (genetic and environmental) confounders shared by siblings.

### Exposure

SGA was defined as having a birth weight for gestational age less than the 10th percentile, according to the ultrasound-based sex-specific Swedish reference curve for normal fetal growth [24]. Gestational age was defined according to ultrasound measurements early in the second trimester or by gestational age estimated from information of the last menstrual period. Early second trimester measurements of fetal dimensions have been offered to all pregnant women in Sweden since 1990 and are performed in some 95% of pregnancies [25]. If no ultrasound data were available, we calculated gestational age based on last menstrual period. SGA was further divided into severe SGA (<3rd percentile) and moderate SGA (3rd to <10th percentile).

### Outcome

The primary outcome measure was all-cause mortality between 28 days and <18 years of age. The secondary outcome measure was cause-specific mortality, focusing on the most common underlying causes of death during childhood: infection, injury, cancer, and neurologic disease [26]. About 96% of deceased individuals have a recorded cause of death in the Cause of Death Register [20]. The specific ICD codes used to classify the underlying causes of death are presented in S1 Table. In an evaluation of deaths occurring among individuals at the age of 0–44 years, the underlying cause of death recorded in the Cause of Death Register (used for this study) was confirmed in 98% of the cases, using medical information from patient charts [27].

### Covariates

Information about maternal education (<10 years, 10–11 years, 12 years, 13–14 years, ≥15 years, or unknown) was derived from the Register of Education, and information about maternal country of birth (Nordic versus non-Nordic country) from the Total Population Register. Other information was gained from the MBR [28]. Preterm births were defined as <37 (and term births as ≥37) completed gestational weeks at the time of delivery. We also obtained information on maternal age, maternal parity (1, 2–3, or ≥4), sex of child, calendar period of birth (1973–1976, 5-year intervals from 1977 to 2006, or 2007–2012), and mode of delivery (vaginal or cesarean delivery). For children born during 1992–2012 (*n* = 1,986,114), we obtained additional information on maternal smoking during pregnancy [29] and body mass index (BMI) in early pregnancy [30,31]. Information on maternal weight for calculating BMI is based on measured maternal weight, and 90% of maternal weight measurements were performed during the first trimester [30].

### Statistical analysis

In the population cohort, we first calculated standardized mortality rates (SMRs, per 10,000 person-years) for all-cause and cause-specific mortality across different ages at follow-up (28 days to <1 year, 1 year to <5 years, 5 years to <10 years, and 10 years to <18 years) in severe SGA, moderate SGA, and non-SGA births separately. Because the mortality rates varied greatly during the 40-year study period, SMRs were estimated using the method of direct standardization by calendar period of birth. The accumulated person-time during the entire follow-up of the population cohort was used as the standard.

We examined the association between SGA and childhood mortality in the population and sibling cohorts. In the population-based analyses we compared mortality risk in children born SGA to that of children not born SGA (the reference group), whereas in the sibling-based analyses we compared mortality risk in children born SGA to that of their non-SGA siblings. In the sibling-based analyses only sibling pairs that were discordant on both exposure (i.e., SGA) and outcome (i.e., mortality hazard) contributed to the estimates. Using attained age at follow-up as the underlying time scale, we derived hazard ratios (HRs) and 95% confidence intervals (CIs) for mortality from ordinary and stratified Cox proportional hazards models in the population- and sibling-based analyses, respectively. Given the observed different SMRs across age groups and for different underlying causes of death, we estimated the HRs for mortality by age at follow-up (28 days to <1 year, 1 year to <5 years, 5 years to <10 years, and 10 years to <18 years) and by underlying cause of death (infection, injury, cancer, and neurologic disease). We further calculated age-specific HRs for the 4 underlying causes of death.

In addition to calculating estimates of age-specific HRs, we used flexible parametric survival models to assess all-cause and cause-specific mortality in relation to severe and moderate SGA. Such models allow the HRs to change continuously over age at follow-up. A spline with 4 degrees of freedom (3 intermediate knots and 2 knots at each boundary, placed at the quintiles of the distribution of death events) was used for the baseline rate, and a spline with 2 degrees of freedom was used for the time-varying effect. We performed comparable analyses for cause-specific mortality. Because of the similar results obtained from the population- and sibling-based analyses, the flexible parametric models were only performed in the population cohort.

To provide additional detailed information on the change in risk of mortality with different birth weight percentiles, and to assess a potential nonlinear relationship, we performed secondary analyses using restricted cubic splines on birth weight percentile for gestational age, instead of categorization. We applied the spline with 4 knots placed at the 0.05, 0.35, 0.65, and 0.95 quantiles of the distribution of outcome events. HRs were estimated in both the population and within-sibling analyses using the 50th percentile as the reference.

In all population-based analyses we adjusted for maternal age, parity, education, and country of birth, and the child’s sex and calendar period of birth. In the sibling-based analyses we adjusted for maternal age and the child’s sex. We did not additionally adjust for parity in the sibling-based analyses as it is highly correlated with maternal age for siblings.

### Additional analyses

To assess the potential effect modification of gestational age, we performed an additional analysis of overall childhood mortality by adding an interaction term between SGA and gestational age. Without assuming a linear relationship, we applied restricted cubic splines on gestational age, and placed 4 knots at the 0.05, 0.35, 0.65, and 0.95 quantiles of the distribution of outcome events [32]. Age-varying HRs were predicted and visualized thereafter. With great interest in term SGA births (as fewer neonatal complications are expected), we further performed a subgroup analysis by restricting the analyses to 3,619,514 term live births in the population-based analysis and 2,597,959 term live births in the sibling-based analysis. Children born with severe SGA are more likely to have a cesarean delivery, and the mode of delivery might influence a child’s immune development through, for example, a differential microflora, and, consequently, its risk of diseases and mortality [33]. We therefore performed a subgroup analysis by mode of delivery for all-cause and cause-specific mortality. As the mortality rate varied greatly over the study period, we also stratified the analyses by calendar period of birth, roughly by decade (1973–1981, 1982–1991, 1992–2001, and 2002–2012).

Although the sibling-based analysis could control for shared familial (genetic as well as in utero and childhood environmental) factors, it could not control for factors that vary between pregnancies. In another analysis, we additionally adjusted for maternal smoking during pregnancy and BMI in early pregnancy among children born between 1992 and 2012. Smoking status was divided into no smoking, smoking, or unknown (4.4% with missing data). BMI was calculated based on the height and weight of the pregnant woman at the first antenatal check-up. To properly address the nonlinear relationship between maternal BMI and offspring mortality, we applied a restricted cubic spline (also 4 knots) to maternal BMI.

We defined statistical significance for HRs as 95% CIs that do not include 1.0. Data were processed by SAS 9.4 (SAS Institute) and analyzed in STATA 14.2 (StataCorp).

### Ethics

The study was approved by the Regional Ethical Review Board in Stockholm, Sweden (No. 2013/2192-32). Because of the strict register-based nature of the study, informed consent was waived [34].

## Results

### Background data

Maternal and infant characteristics at the time of birth are shown in Table 1. Among all live births included in the population cohort, 80,924 were born with severe SGA (2.1%) and 216,037 with moderate SGA (5.7%). Preterm births accounted for 4.6% of all births, and 16.3% and 6.0% for severe and moderate SGA births, respectively.

**Table 1:** Maternal and child characteristics at the time of birth for all live births without major malformations during 1973–2012 in Sweden by birth weight for gestational age.

Characteristic	Birth weight <3rd percentile *n* (%)	Birth weight 3rd to <10th percentile *n* (%)	Birth weight ≥10th percentile *n* (%)
**Total**	80,924	216,037	3,498,642
**Maternal age (years)**			
** **<20	4,353 (5.4)	10,554 (4.9)	103,579 (3.0)
** **20–24	20,798 (25.7)	54,592 (25.3)	697,728 (19.9)
** **25–29	26,382 (32.6)	74,244 (34.4)	1,223,432 (35.0)
** **30–34	18,850 (23.3)	51,587 (23.9)	990,387 (28.3)
** **≥35	10,541 (13.0)	25,060 (11.6)	483,516 (13.8)
**Maternal parity**			
** **1	47,409 (58.6)	122,068 (56.5)	1,450,940 (41.5)
** **2–3	29,472 (36.4)	84,043 (38.9)	1,840,088 (52.6)
** **≥4	4,043 (5.0)	9,926 (4.6)	207,614 (5.9)
**Maternal education (years)**			
** **<10	14,871 (18.4)	35,830 (16.6)	428,811 (12.3)
** **10–11	26,383 (32.6)	65,694 (30.4)	939,308 (26.8)
** **12	12,225 (15.1)	35,500 (16.4)	646,326 (18.5)
** **13–14	9,649 (11.9)	27,384 (12.7)	506,245 (14.5)
** **≥15	14,400 (17.8)	43,775 (20.3)	895,066 (25.6)
** **Missing	3,396 (4.2)	7,854 (3.6)	82,886 (2.4)
**Mother’s country of birth**			
** **Nordic*	69,989 (86.5)	183,623 (85.0)	3,092,554 (88.4)
** **Non-Nordic	10,378 (12.8)	31,004 (14.4)	392,317 (11.2)
** **Missing	557 (0.7)	1,410 (0.7)	13,771 (0.4)
**Calendar period of childbirth**			
** **1973–1976	14,595 (18.0)	33,136 (15.3)	344,832 (9.9)
** **1977–1981	13,769 (17.0)	33,174 (15.4)	398,620 (11.4)
** **1982–1986	10,445 (12.9)	27,221 (12.6)	401,239 (11.5)
** **1987–1991	11,281 (13.9)	29,135 (13.5)	492,042 (14.1)
** **1992–1996	8,602 (10.6)	24,264 (11.2)	469,394 (13.4)
** **1997–2001	6,507 (8.0)	18,654 (8.6)	382,505 (10.9)
** **2002–2006	6,821 (8.4)	21,001 (9.7)	434,798 (12.4)
** **2007–2012	8,904 (11.0)	29,452 (13.6)	575,212 (16.4)
**Sex of child**			
** **Male	40,469 (50.0)	107,074 (49.6)	1,791,583 (51.2)
** **Female	40,455 (50.0)	108,963 (50.4)	1,707,059 (48.8)
**Mode of delivery**			
** **Vaginal delivery	56,980 (70.4)	185,086 (85.7)	3,094,500 (88.5)
** **Cesarean section	23,944 (29.6)	30,951 (14.3)	404,142 (11.5)
**Preterm birth (<37 gestational weeks)**			
** **Yes	13,209 (16.3)	12,961 (6.0)	149,919 (4.3)
** **No	67,715 (83.7)	203,076 (94.0)	3,348,723 (95.7)

*Includes Denmark, Finland, Iceland, Norway, and Sweden.

During the follow-up (median 18 years), there were 10,838 deaths in the population cohort. Crude mortality rates per 10,000 person-years were 5.32 in children born with severe SGA, 2.76 in children born with moderate SGA, and 1.93 in non-SGA children. There were 1,572 deaths among the informative sibling pairs in the sibling cohort. More deaths were observed among SGA children who were born during earlier calendar periods compared to later calendar periods.

### Mortality across age at follow-up

**Fig 1:**
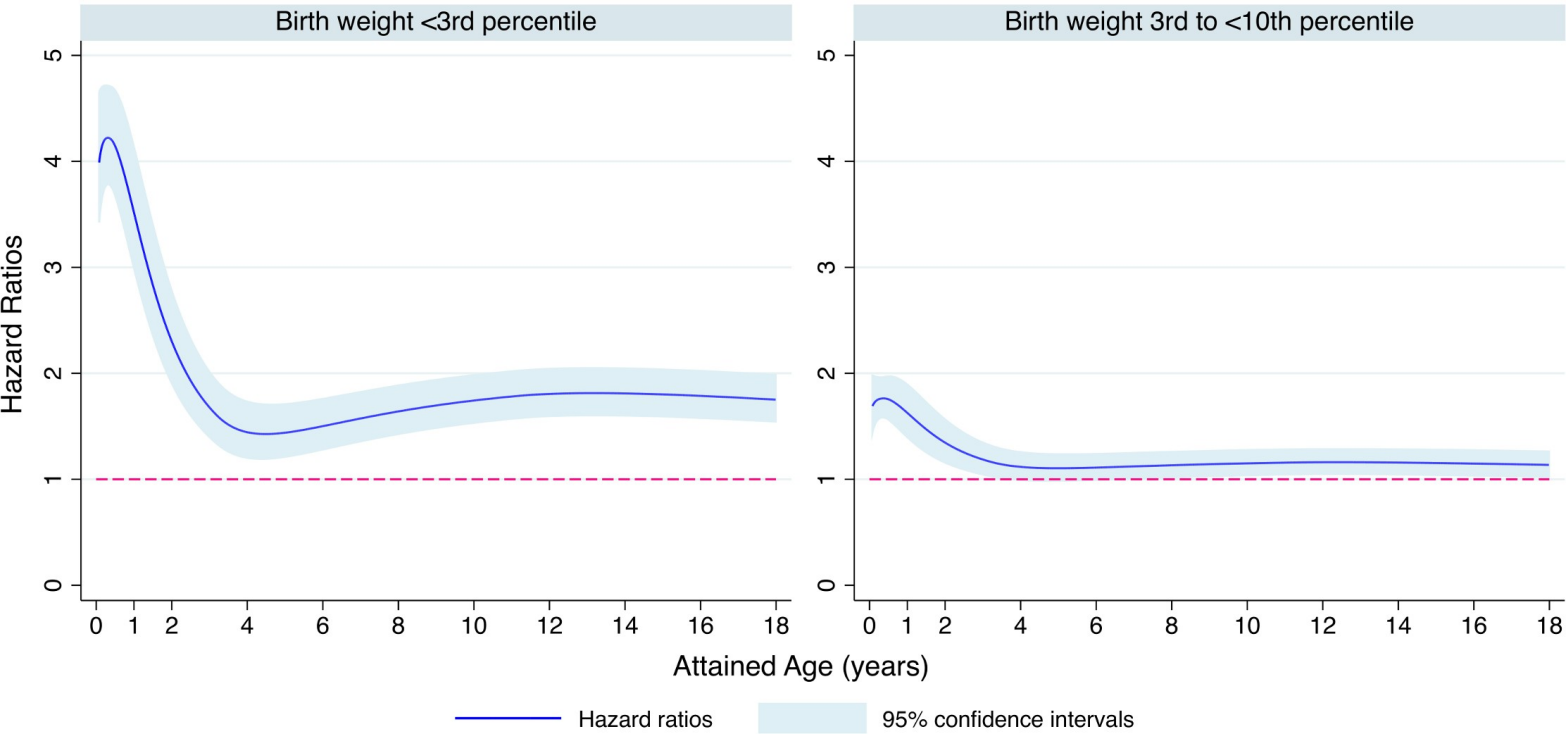
Association of small for gestational age (SGA) with childhood all-cause mortality (age 28 days to <18 years) by attained age in the population analysis. Time-varying hazard ratios (blue line) and 95% confidence intervals (shading) were derived from the flexible parametric survival model, allowing the effect of SGA to vary over time. A spline with 4 degrees of freedom (3 intermediate knots and 2 knots at each boundary, placed at the quintiles of the distribution of events) was used for the baseline rate, and 2 degrees of freedom was used for the time-varying effect. Hazard ratios were adjusted for maternal age, maternal education level (<10 years, 10–11 years, 12 years, 13–14 years, ≥15 years, or unknown), maternal country of birth (Nordic or non-Nordic country), maternal parity (1, 2–3, or ≥4), the child’s sex, and calendar period of birth (1973–1976, 5-year intervals from 1977 to 2006, or 2007–2012).

Overall and age-specific all-cause mortality rates were higher in children born with severe SGA than in children born with moderate SGA or non-SGA in both the population and sibling analyses (Table 2). Compared with non-SGA children, children born with severe SGA had an increased overall mortality in the population-based (HR = 2.58, 95% CI = 2.38–2.80) and sibling-based (HR = 2.61, 95% CI = 2.19–3.10) analyses. Such associations were largely confirmed when using birth weight percentile for gestational age as a continuous variable (S4 Fig). The associations were strongest between 28 days and <1 year of age (HR = 4.46, 95% CI = 3.98–5.00, in the population-based analysis and HR = 3.41, 95% CI = 2.67–4.36, in the sibling-based analysis; Table 2). Severe SGA was associated with an increased mortality rate in all age groups, but the magnitude declined with increasing age. Similar but weaker overall associations were found for moderate SGA in both the population-based (HR = 1.37, 95% CI = 1.28–1.47) and sibling-based (HR = 1.38, 95% CI = 1.22–1.56) analyses. Similar to severe SGA, these associations were also strongest between 28 days and <1 year of age and declined thereafter. Moderate SGA was not associated with increased mortality beyond 10 years of age. The age-varying HRs for mortality in severe and moderate SGA were further confirmed in flexible parametric modeling (Fig 1).

**Table 2:** Association of small for gestational age (SGA) with childhood mortality by age group in a cohort study of all live births without major malformations during 1973–2012 in Sweden.

Age group	Population analysis			Sibling analysis		
	Number of children	Number of events	HR (95% CI)*	Number of children^†^	Number of events^†^	HR (95% CI)^‡^
**28 days to <18 years**						
Birth weight for gestational age (percentiles)						
** **<3rd	80,924	642	2.58 (2.38–2.80)	688	388	2.61 (2.19–3.10)
** **3rd to <10th	216,037	863	1.37 (1.28–1.47)	1,279	523	1.38 (1.22–1.56)
** **≥10th	3,498,642	9,333	1.0	2,808	661	1.0
**28 days to <1 year**						
Birth weight for gestational age (percentiles)						
** **<3rd	80,924	336	4.46 (3.98–5.00)	369	210	3.41 (2.67–4.36)
** **3rd to <10th	216,037	365	1.86 (1.66–2.07)	563	231	1.73 (1.43–2.09)
** **≥10th	3,498,642	3,104	1.0	1,356	274	1.0
**1 year to <5 years**						
Birth weight for gestational age (percentiles)						
** **<3rd	80,241	126	2.17 (1.81–2.59)	139	82	2.86 (1.94–4.21)
** **3rd to <10th	214,774	188	1.27 (1.10–1.48)	297	116	1.39 (1.06–1.82)
** **≥10th	3,484,292	2,168	1.0	620	142	1.0
**5 years to <10 years**						
Birth weight for gestational age (percentiles)						
** **<3rd	73,177	64	1.65 (1.28–2.12)	55	32	2.03 (1.11–3.69)
** **3rd to <10th	192,066	119	1.24 (1.03–1.50)	159	70	1.32 (0.92–1.87)
** **≥10th	3,053,878	1,368	1.0	323	81	1.0
**10 years to <18 years**						
Birth weight for gestational age (percentiles)						
** **<3rd	65,413	116	1.49 (1.24–1.80)	132	64	1.39 (0.95–2.03)
** **3rd to <10th	167,594	191	1.00 (0.86–1.16)	273	104	0.91 (0.70–1.19)
** **≥10th	2,578,060	2,693	1.0	517	169	1.0

*HRs in the population analysis were adjusted for maternal age, maternal education level (<10 years, 10–11 years, 12 years, 13–14 years, ≥15 years, or unknown), maternal country of birth (Nordic or non-Nordic country), maternal parity (1, 2–3, or ≥4), the child’s sex, and calendar period of birth (1973–1976, 5-year intervals from 1977 to 2006, or 2007–2012).

^†^In the within-sibling analysis, number of births represents the number of informative siblings, namely siblings who were discordant for both exposure (SGA versus non-SGA) and outcome (death or alive) who contributed to the risk estimates, although all children with siblings were included for analysis.

^‡^HRs in the sibling analyses were adjusted for maternal age and the child’s sex.

HR, hazard ratio.

### Mortality by underlying cause of death

The most common cause of death during follow-up was injury (Table 3). However, the strongest association of severe SGA with cause-specific mortality was noted for death due to infection (HR = 3.19 in the population-based analysis and HR = 4.24 in the sibling-based analysis), followed by neurologic disease (HR = 2.22 in the population-based analysis and HR = 1.97 in the sibling-based analysis) and injury (HR = 1.32 in the population-based analysis and HR = 1.43 in the sibling-based analysis), although some associations were of borderline significance in the sibling-based analyses. In both the population and sibling analyses, moderate SGA was associated with a higher risk of death caused by infection (HR = 1.63 in the population-based analysis and HR = 1.49 in the sibling-based analysis) and neurologic disease (HR = 1.55 in the population-based analysis and HR = 2.14 in the sibling-based analysis). Severe and moderate SGA did not influence risk of cancer-related mortality.

**Table 3:** Association of small for gestational age (SGA) with childhood mortality (age 28 days to <18 years) by underlying cause of death in a cohort study of all live births without major malformations during 1973–2012 in Sweden.

Cause of death	Population analysis			Sibling analysis		
	Number of children	Number of events	HR (95% CI)*	Number of children^†^	Number of events^†^	HR (95% CI)^‡^
**Infection**						
Birth weight for gestational age (percentiles)						
** **<3rd	80,924	74	3.19 (2.51–4.06)	73	42	4.24 (2.39–7.53)
** **3rd to <10th	216,037	96	1.63 (1.32–2.01)	153	62	1.49 (1.03–2.16)
** **≥10th	3,498,642	851	1.0	375	74	1.0
**Injury**						
Birth weight for gestational age (percentiles)						
** **<3rd	80,924	113	1.32 (1.10–1.60)	117	58	1.43 (0.96–2.13)
** **3rd to <10th	216,037	223	1.08 (0.94–1.24)	323	119	1.00 (0.78–1.29)
** **≥10th	3,498,642	2,871	1.0	610	176	1.0
**Cancer**						
Birth weight for gestational age (percentiles)						
** **<3rd	80,924	50	1.23 (0.93–1.63)	66	30	1.47 (0.82–2.64)
** **3rd to <10th	216,037	86	0.84 (0.68–1.05)	133	56	1.02 (0.69–1.50)
** **≥10th	3,498,642	1,474	1.0	264	81	1.0
**Neurologic disease**						
Birth weight for gestational age (percentiles)						
** **<3rd	80,924	37	2.22 (1.59–3.09)	38	20	1.97 (0.97–4.01)
** **3rd to <10th	216,037	66	1.55 (1.20–2.01)	92	47	2.14 (1.34–3.42)
** **≥10th	3,498,642	631	1.0	178	40	1.0

*HRs in the population analysis were adjusted for maternal age, maternal education level (<10 years, 10–11 years, 12 years, 13–14 years, ≥15 years, or unknown), maternal country of birth (Nordic or non-Nordic country), maternal parity (1, 2–3, or ≥4), the child’s sex, and calendar period of birth (1973–1976, 5-year intervals from 1977 to 2006, or 2007–2012).

^†^In the within-sibling analysis, number of births represents the number of informative siblings, namely siblings who were discordant for both exposure (SGA versus non-SGA) and outcome (death or alive) who contributed to the risk estimates, although all children with siblings were included for analysis.

^‡^HRs in the sibling analyses were adjusted for maternal age and the child’s sex.

HR, hazard ratio.

**Fig 2:**
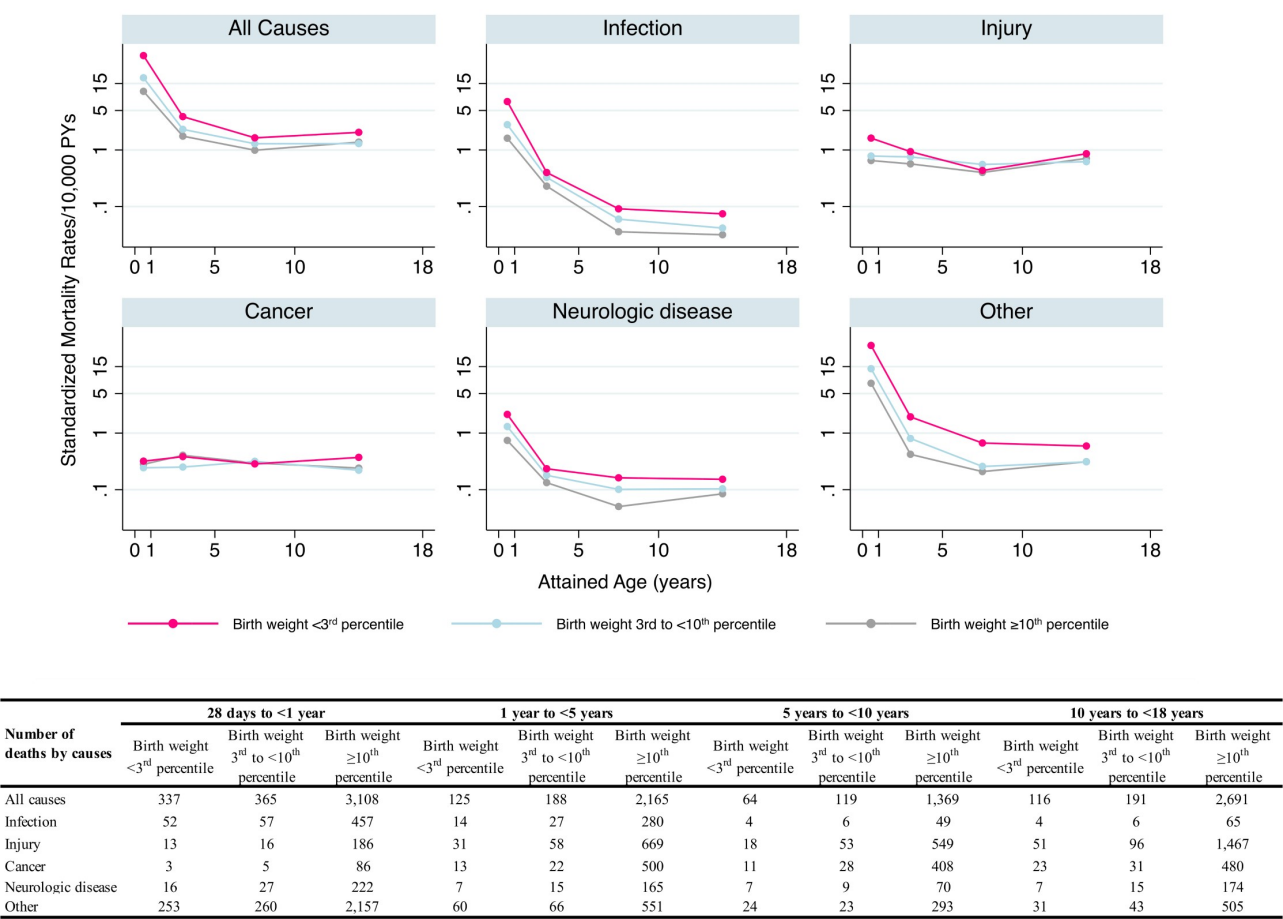
Standardized mortality rates from 28 days to <18 years of age in all live births without major malformations during 1973–2012 in Sweden by birth weight for gestational age and underlying cause of death. Mortality rates by attained age (28 days to <1 year, 1 year to <5 years, 5 years to <10 years, and 10 years to <18 years) were standardized by calendar period and plotted in log scale. PYs, person-years.

Overall and cause-specific mortality rates across age at follow-up are displayed in Fig 2. The associations between severe SGA and risk of death due to infection and neurologic disease were strongest during the first year of life, but risk of death due to infection also remained high later in childhood (S1 Fig). No clear association was noted for severe SGA and death from cancer. Largely similar patterns, but weaker associations, were noted for moderate SGA (S1 and S2 Figs). Similar patterns were observed when separately calculating the age-specific associations for different causes of death in the population-based analysis (S2 Table).

### Additional analyses

Slightly stronger HRs for mortality were suggested for preterm SGA, particularly for severe SGA, despite wide confidence intervals (S3 Fig). Subgroup analysis restricting the age- and cause-specific mortality analyses to term live births (≥37 gestational weeks) rendered largely unchanged results (S3 and S4 Tables). We also stratified analyses by mode (S5 Table) and year of delivery (S6 Table). Overall and infection-related mortality were generally higher in children with severe and moderate SGA delivered by cesarean section than in those with vaginal delivery (S5 Table). The associations of severe and moderate SGA with all-cause mortality from 28 days to <1 year of age were stronger in the most recent birth cohorts compared with earlier birth cohorts (S6 Table). Further adjustment for maternal smoking during pregnancy and BMI in early pregnancy only marginally influenced the results (S7 Table).

## Discussion

In this population study of more than 3.7 million children we found that severe SGA (<3rd percentile) was associated with increased mortality from 28 days to <18 years of life. Moderate SGA (3rd to <10th percentile) was associated with more modestly increased mortality within the first 10 years of life. The similar results between the population- and sibling-based analyses argue against strong confounding by familial factors. Given the high prevalence of SGA, our findings have important public health implications and underline the need to tackle long-term consequences of SGA.

Expectedly, severe SGA was associated with an increased risk of postneonatal mortality [8,9]. Our study confirms previous findings [8,9,16] and contributes additional evidence in several important ways. First, the large sample size allowed us to calculate precise relative risk estimates (HR = 2.58, 95% CI = 2.38–2.80, in the population-based analysis). Second, we were able to differentiate between severe and moderate SGA. Third, we excluded children with malformations from our analyses. Fourth, we used a sibling control design, which, by design, controls for genetic and environmental factors shared by siblings. Thus, access to data on siblings allowed us to eliminate the influence of certain intrafamilial characteristics, including maternal traits that may otherwise be difficult to measure. We found similar HRs for childhood mortality beyond age 28 days in the population and sibling analyses, suggesting that the association of SGA with childhood mortality is unlikely to be dependent on familial factors. It is also well recognized that preterm birth, often linked to SGA, contributes to long-term health consequences, including mortality [35,36]. However, in a sensitivity analysis we restricted the comparisons to term births and found largely unchanged results, alleviating the potential concern that the excess mortality in children born SGA is due to underlying prematurity alone.

We were able to examine mortality at different ages during childhood, where some distinct patterns emerged. With age, the relative risk of mortality for severe and moderate SGA decreased: for severe SGA, the decrease went from a HR of 4.46 up until age 1 year to a HR of 1.49 at ages between 10 and <18 years in the population-based analysis. This finding is consistent with data from Denmark, where SGA less than the 10th percentile was associated with increased mortality that decreased over time (HR = 3.47 for <2 years including the neonatal period, 1.70 for 2–5 years, 1.42 for 6–13 years, and 1.34 for 14–19 years) [16]. As shown in the flexible parametric modeling, our findings further highlight the greater mortality rate under 4 years. While childhood mortality is decreasing, a recent UNICEF report warns that the Millennium Development Goal to reduce the under-5 mortality rate by two-thirds may not be met until 2026 [11]. Maternal antenatal care, in order to globally reduce the risk of SGA birth, is key to achieving that goal [11].

The large sample size also allowed us to examine causes of death and to speculate on possible mechanisms for the noted positive association between SGA and childhood mortality. We focused on infection, injury, cancer, and neurologic disease, which are the most common underlying causes of death during childhood [26] and reflect different mechanisms of action. For severe SGA, we noted an excess mortality for all causes except cancer in early childhood, but only for infection and neurologic disease between 10 and <18 years of age. Wennerström et al. also reported an increased risk of death from infection throughout childhood (HR = 1.52); however, they did not report risk estimates for neurologic disease [16]. We found largely a null association between SGA and death from cancer, except for at age 10 to <18 years. This result was expected given the null results of the Wennerström et al. study, which also reported a positive association between large for gestational age and cancer risk [16].

Our study showed consistently higher HRs for the association of SGA with mortality in the latest calendar period, although risk of death at 10 to <18 years could not be calculated for children born in 2002–2012 because of the unavailable data. For instance, children born with severe SGA in 1973–1981 were shown to have a 1.92-fold increased risk of death between 5 and 10 years of age (95% CI = 1.38–2.68), whereas children born with severe SGA in 2002–2012 had a 2.55-fold increased risk (95% CI = 1.04–6.27), compared with non-SGA children born during the respective time periods (S6 Table). Similarly, stronger associations were seen for other age groups in children born after 2002. The mechanisms for this are not clear. We speculate that this may partly be explained by a greater improvement of long-term survival in children born non-SGA than in those born with severe SGA during the study period. Another possible explanation may be that children born SGA today are more likely to survive the neonatal period compared with children born SGA in the past.

### Strengths and limitations

The free and standardized medical care in Sweden [37] minimizes selection bias. We therefore believe that our data are representative of the whole population and that our findings can be generalized to similar populations. We used nationwide registers to define both exposures and outcomes. These registers contain prospectively collected, high-quality data on healthcare, which minimize the risk of differential measurement biases.

We adjusted for a number of potentially important confounders (e.g., maternal age [38], maternal education level [39], maternal country of birth [39], parity, infant sex, and calendar year of birth [39]). We used a sibling control design to further minimize the influence of unmeasured confounding (such as by feto-maternal factors) and found similar results in the population- and sibling-based analyses. In a subset of the sibling-based analyses, we further adjusted for maternal smoking during pregnancy and BMI in early pregnancy, 2 exposures that might differ between pregnancies, and found comparable results. The similar results obtained from this analysis further alleviated concerns of residual confounding. Furthermore, because the origin of term SGA and preterm SGA may differ, we also performed sensitivity analyses restricted to term live births and again found similar results.

We acknowledge a number of limitations. Although we have to our knowledge the largest study sample so far addressing the research question, some subgroup analyses, especially the analysis of cause-specific mortality, had limited statistical power in the sibling-based analysis. The proportion of children born SGA was 7.8% in our study, which is lower than the expected 10%. This is probably due to population changes over time [40]. The Swedish reference curve for normal fetal growth is based on pregnancies until the early 1990s [24]. A Swedish study reported an increase in birth weight from 1992 to 2001, and that the increase in large for gestational age births was explained by concurrent increases in maternal BMI and decreases in maternal smoking [41]. Since 2001, maternal obesity has continued to increase and maternal smoking to decrease in Sweden [42]. Other limitations include potential misclassification, missing data on some covariates, and that not all ICD codes used in our study have been validated. Finally, we did not have data on the precise indication for cesarean section and could therefore not assess whether the indication for cesarean section or the surgery itself modified the association of SGA with childhood mortality.

### Mechanisms

While a host of different causes of death were overrepresented in SGA children, deaths from infection may be particularly important as these represent an important cause of death in absolute numbers and because children with severe SGA had a 3–4 times higher risk of death due to infection, compared both with population controls and with their siblings. This finding was also seen when restricting the analysis to term live births, suggesting that the association was not driven by preterm birth.

Fetal growth restriction may potentially influence organ development and maturation of the immune system, leading to an altered risk of infections [43,44]. For instance, it has been shown that the risk of perinatal sepsis increases with growth restriction [45]. Few studies, however, have specifically examined the associations of severe and moderate SGA with the risk of fatal infection beyond the first year of life. SGA may be linked to lower levels of certain cytokines (e.g., interleukin 1 beta) [46], which are involved in the inflammatory response in infection. Finally, children born SGA are more likely to receive neonatal intensive care [47], and it has been shown that early exposure to intensive care, with frequent use of antibiotics, may have an impact on immune development [48].

We also found a positive association between SGA and death from neurologic disease, which confirms a Danish study reporting that the incidence of epilepsy in the first 5 years of life was correlated with birth weight by gestational age [49]. In children with severe SGA we also noted a small excess risk of death from injury. We speculate that this may potentially be due to impaired neuropsychological development and thereby deficient cognitive function [50]. Although the absolute risk increase associated with SGA was smaller, compared to deaths due to infection and neurologic disease, the fact that injury is the leading cause of childhood mortality worldwide makes this finding both relevant and important.

### Clinical implications

SGA cannot be reversed, and hence primary preventive strategies are needed to decrease risk of intrauterine growth restriction. Such measures include smoking cessation and nutritional support in pregnant woman. Antenatal detection of SGA using symphysis-fundal height measurement and ultrasound (and where available Doppler velocimetry), followed by targeted interventions [51], may reduce the risk of not only perinatal death but also death throughout childhood. Furthermore, fetal growth in SGA should be carefully monitored through repeated ultrasound of fetal dimensions. Optimal obstetrical and neonatal care is necessary to reduce risks of asphyxia-related neonatal complications.

Our findings provide important insights on associations between SGA and causes of death in childhood. Along with the global call, active follow-up on SGA children up to 4 years of age might further reduce the mortality rate under 5 years, even in high-income countries. Finally, we want to highlight the excess risk of death due to infection in children born SGA. Even if the absolute risk is low, death caused by infectious disease can often be prevented through timely detection and the early use of appropriate antibiotics. More research is however needed to identify subgroups for which interventions may be both clinically effective and cost-effective.

### Conclusion

In conclusion, population- and sibling-based analyses show that SGA and fetal growth restriction are associated with increased mortality throughout childhood.

## Supporting information

S1 Fig Association of severe small for gestational age (SGA; birth weight for gestational age <3rd percentile) with the risk of childhood cause-specific mortality (age 28 days to <18 years) by attained age in the population analysis. (DOCX)

S2 Fig Association of moderate small for gestational age (SGA; birth weight for gestational age from 3rd to <10th percentile) with the risk of childhood cause-specific mortality (age 28 days to <18 years) by attained age in the population analysis. (DOCX)

S3 Fig Association of moderate small for gestational age (SGA) with the risk of childhood all-cause mortality (age 28 days to <18 years) across gestational age in a cohort study of all live births without major malformations during 1973–2012 in Sweden. (DOCX)

S4 Fig Association of birth weight percentile for gestational age with childhood cause-specific mortality (age 28 days to <18 years) in population and sibling analyses. (DOCX)

S1 STROBE STROBE checklist of items that should be included in reports of cohort studies. (DOC)

S1 Table ICD-8/9/10 codes for causes of death and malformations. (DOCX)

S2 Table Association of small for gestational age (SGA) with the risk of childhood mortality (age 28 days to <18 years) by underlying cause of death and age at follow-up in a cohort study of all live births without major malformations during 1973–2012 in Sweden. (DOCX)

S3 Table Association of small for gestational age (SGA) with the risk of childhood mortality by age group in a cohort study of all live births without major malformations during 1973–2012 in Sweden: Analysis restricted to term births (≥37 gestational weeks). (DOCX)

S4 Table Association of small for gestational age (SGA) with the risk of childhood mortality (age 28 days to <18 years) by underlying cause of death in a cohort study of all live births without major malformations during 1973–2012 in Sweden: Analysis restricted to term births (≥37 gestational weeks). (DOCX)

S5 Table Association of small for gestational age (SGA) with the risk of childhood mortality (age 28 days to <18 years) by underlying cause of death and modality of delivery in a cohort study of all live births without major malformations during 1973–2012 in Sweden. (DOCX)

S6 Table Association of small for gestational age (SGA) with the risk of childhood mortality (age 28 days to <18 years) by age at follow-up, and calendar period of birth in a cohort study of all live births without major malformations during 1973–2012 in Sweden. (DOCX)

S7 Table Association of small for gestational age (SGA) with the risk of childhood mortality by age group in a cohort study of all live births without major malformations during 1992–2012 in Sweden: Analyses adjusted for maternal smoking and body mass index (BMI). (DOC)

## Abbreviations

HR: hazard ratio
ICD: International Classification of Diseases
MBR: Medical Birth Register
PIN: personal identity number
SGA: small for gestational age
SMR: standardized mortality rate
