# Supplementary material for: Small for gestational age and risk of childhood mortality: A Swedish population study
Source: PLoS Med. 2018 Dec 18;15(12):e1002717. doi: 10.1371/journal.pmed.1002717 (PMC6298647; doi:10.1371/journal.pmed.1002717)

**S2 Fig. Association of moderate small for gestational age (SGA; birth weight for gestational age from 3^rd^ to <10^th^ percentile) with the risk of childhood cause-specific mortality (age from 28 days to <18 years) by attained age in the population analysis.** Time-varying hazard ratios were derived from the flexible parametric survival model, allowing the effect of SGA to vary over time. A spline with 4 df (3 intermediate knots and 2 knots at each boundary, placed at quintiles of distribution of events) was used for the baseline rate, and 2 df was used for the time-varying effect. Hazard ratios were adjusted for maternal age, maternal education level (<10 years, 10-11 years, 12 years, 13-14 years, ≥15 years, or unknown), maternal country of birth (Nordic or non-Nordic country), maternal parity (1, 2-3, or ≥4), child’s sex, and calendar period of birth (1973-1976, every 5 years thereafter, or 2007-2012).


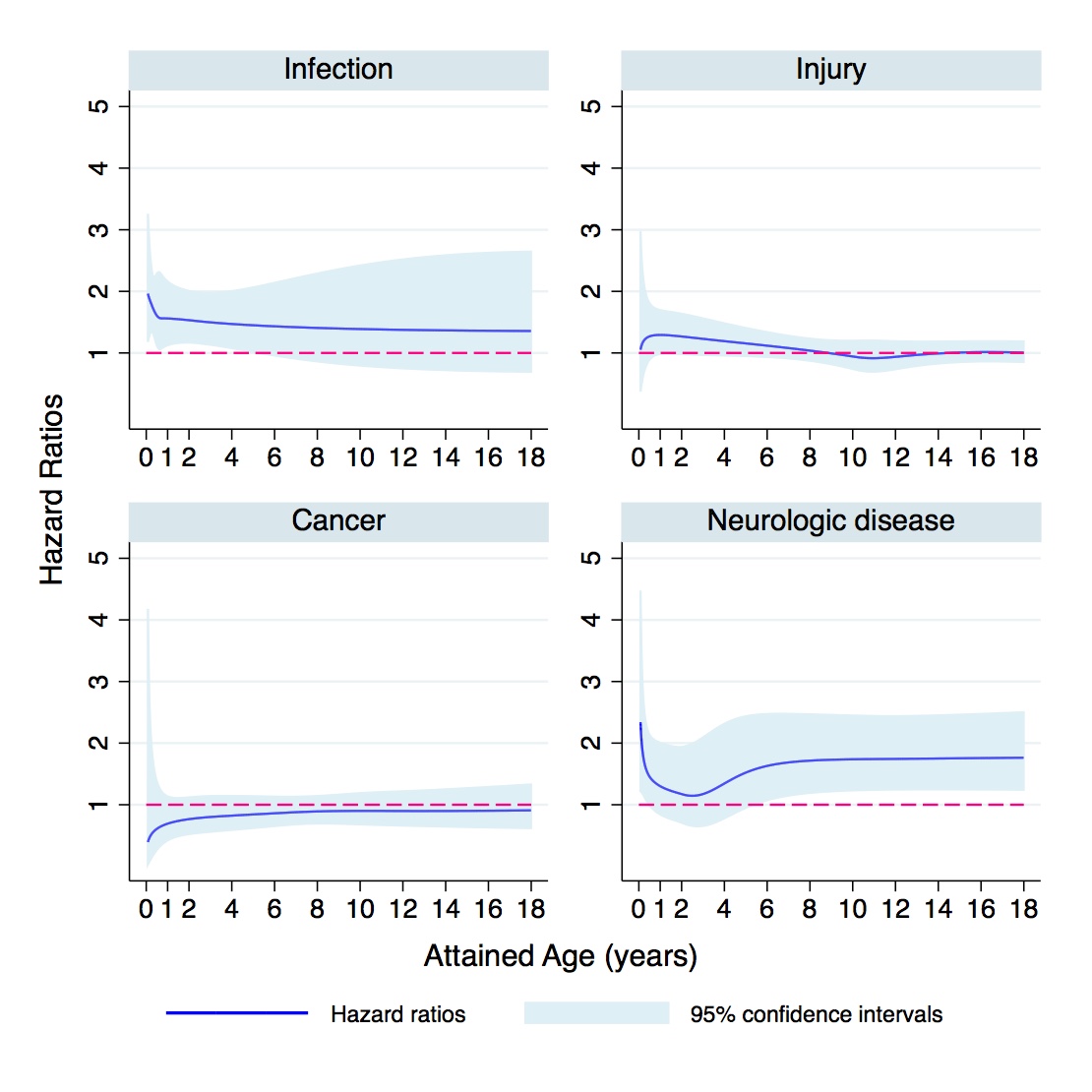

Supplement: S2 Fig — (DOCX) [file pmed.1002717.s002.docx]
