# Supplementary material for: Small for gestational age and risk of childhood mortality: A Swedish population study
Source: PLoS Med. 2018 Dec 18;15(12):e1002717. doi: 10.1371/journal.pmed.1002717 (PMC6298647; doi:10.1371/journal.pmed.1002717)
Supplement: S3 Fig — (DOCX) [file pmed.1002717.s003.docx]

**S3 Fig. Association of moderate small for gestational age (SGA) with the risk of childhood all-cause mortality (age from 28 days to <18 years) across gestational age, a cohort study of all live births without major malformations during 1973-2012 in Sweden.** We first applied restricted cubic splines on gestational age, and placed four knots placed at 0.05, 0.35, 0.65 and 0.95 quantiles of the distribution of outcome events. We then added an interaction term between SGA and the splined gestational age, and reported the varying hazard ratios (HRs) of childhood all-cause mortality among SGA births over gestational age. HRs in the population analysis were adjusted for maternal age, maternal education level (<10 years, 10-11 years, 12 years, 13-14 years, ≥15 years, or unknown), maternal country of birth (Nordic or non-Nordic country), maternal parity (1, 2-3, or ≥4), child’s sex, and calendar period of birth (1973-1976, every 5 years thereafter, or 2007-2012). HRs in the sibling analyses were adjusted for maternal age and child’s sex.
