# Supplementary material for: Small for gestational age and risk of childhood mortality: A Swedish population study
Source: PLoS Med. 2018 Dec 18;15(12):e1002717. doi: 10.1371/journal.pmed.1002717 (PMC6298647; doi:10.1371/journal.pmed.1002717)
Supplement: S4 Fig — (DOCX) [file pmed.1002717.s004.docx]

**S4 Fig. Association of birth weight percentile for gestational age with childhood cause-specific mortality (age from 28 days to <18 years) in population and sibling analyses.** We applied restricted cubic spline on birth weight percentile with four knots placed at 0.05, 0.35, 0.65 and 0.95 quantiles of the distribution of outcome events. Hazard ratios in population analysis were adjusted for maternal age, maternal education level (<10 years, 10-11 years, 12 years, 13-14 years, ≥15 years, or unknown), maternal country of birth (Nordic or non-Nordic country), maternal parity (1, 2-3, or ≥4), child’s sex, and calendar period of birth (1973-1976, every 5 years thereafter, or 2007-2012). Hazard ratios in sibling analysis were adjusted for maternal age and child’s sex.
