# Supplementary material for: Small for gestational age and risk of childhood mortality: A Swedish population study
Source: PLoS Med. 2018 Dec 18;15(12):e1002717. doi: 10.1371/journal.pmed.1002717 (PMC6298647; doi:10.1371/journal.pmed.1002717)
Supplement: S1 Table — (DOCX) [file pmed.1002717.s006.docx]

**S1 Table. ICD-8/9/10 codes for causes of death and malformations**

|  | **ICD-8 codes** | **ICD-9 codes** | **ICD-10 codes** |
| --- | --- | --- | --- |
| **Causes of death^*^** |  |  |  |
| Infection | 000-134,320,380-383, 460-486, 49102, 566, 56700, 56701, 56900, 590, 595, 710, 720 | 000-134, 320, 380-383, 460-466, 480-487, 4911, 566, 5670, 5671, 590, 595, 680-686, 7110, 730, 770, 771 | A00-B99, G00-G02, G042, G06-G07, H66, H700, H701, J00-J22, J32, J350, L00-L04, L08, L303, M00, M01, N10, N11, P23, P35-P39, |
| Injury | 80-93, 95-99 | 80-86, 88-92, 95-99 | V01-Y36, Y85-Y87, Y89 |
| Cancer | 140-209 | 140-209 | C00-C97 |
| Neurologic disease | 330-358 | 330-359 | G10-G99 |
|  |  |  |  |
|  |  |  |  |
|  | **ICD-8 codes** | **ICD-9 codes** | **ICD-10 codes** |
| **Major malformations**^†^ | 740-759, excluding:  745.1, 745.2, 745.4, 747.0, 747.7, 748.3, 750.0, 752.6, 752.8, 752.1, 755.6, 755.7, 755.0, 756.1, 756.9, 757.2 | 740-759, excluding:  744B, 744C, 744E, 747A, 747F, 748D, 750A, 752E, 752F, 754D, 754G, 755A, 755B, 756B, 756X, 757C | Q00-Q99, excluding:  Q170, Q175, Q180, Q181, Q250, Q270, Q314, Q320, Q381, Q523, Q530-Q539, Q650-Q659, Q665-Q669, Q690, Q699, Q703, Q760, Q799, Q825, Q829 |
| ^*^ Underlying cause of death was obtained from the Cause of Death Register.  ^†^ Diagnosis of malformations is derived from the Medical Birth Register (1973-2012) or the Patient Register (in-patient hospital care 1973-2012 or out-patient hospital care 2001-2012) at 0-364 days of life. Minor malformations as defined by the Swedish National Board of Health and Welfare (see <http://www.socialstyrelsen.se/blanketter/Documents/Dnr%2034-3711-2007.pdf>) were excluded from major malformations. | | | |
