# Supplementary material for: Small for gestational age and risk of childhood mortality: A Swedish population study
Source: PLoS Med. 2018 Dec 18;15(12):e1002717. doi: 10.1371/journal.pmed.1002717 (PMC6298647; doi:10.1371/journal.pmed.1002717)
Supplement: S2 Table — (DOCX) [file pmed.1002717.s007.docx]

**S2 Table. Association of small for gestational age (SGA) with the risk of childhood mortality (age from 28 days to <18 years) by underlying cause of death and age at follow-up, a cohort study of all live births without major malformations during 1973-2012 in Sweden.**

| **Cause of death/age groups** | **28 days to <1 year** | | **1 year to <5 years** | | **5 years to <10 years** | | **10 years to <18 years** | |
| --- | --- | --- | --- | --- | --- | --- | --- | --- |
|  | **N children /events** | **HR (95% CI)*^*^*** | **N children /events** | **HR (95% CI)*^*^*** | **N children /events** | **HR (95% CI)*^*^*** | **N children /events** | **HR (95% CI)*^*^*** |
| **Infection** |  |  |  |  |  |  |  |  |
| Birth weight for gestational age (percentiles) |  |  |  |  |  |  |  |  |
| <3^rd^ | 80 924/52 | 4.30 (3.22-5.75) | 80 241/14 | 1.76 (1.03-3.02) | 73 177/4 | 2.84 (1.02-7.94) | 65 413/4 | 2.24 (0.81-6.18) |
| 3^rd^ to <10^th^ | 216 037/57 | 1.83 (1.39-2.42) | 214 774/27 | 1.33 (0.90-1.99) | 192 066/6 | 1.76 (0.75-4.12) | 167 594/6 | 1.37 (0.59-3.17) |
| ≥10^th^ | 3 498 642/456 | 1.0 | 3 484 292/281 | 1.0 | 3 053 878/49 | 1.0 | 2 578 060/65 | 1.0 |
| **Injury** |  |  |  |  |  |  |  |  |
| Birth weight for gestational age (percentiles) |  |  |  |  |  |  |  |  |
| <3^rd^ | 80 924/13 | 2.34 (1.32-4.12) | 80 241/31 | 1.61 (1.12-2.31) | 73 177/18 | 1.10 (0.69-1.76) | 65 413/51 | 1.16 (0.88-1.54) |
| 3^rd^ to <10^th^ | 216 037/16 | 1.15 (0.69-1.93) | 214 774/58 | 1.23 (0.94-1.61) | 192 066/53 | 1.35 (1.02-1.79) | 167 594/96 | 0.90 (0.73-1.11) |
| ≥10^th^ | 3 498 642/184 | 1.0 | 3 484 292/671 | 1.0 | 3 053 878/548 | 1.0 | 2 578 060/1 468 | 1.0 |
| **Cancer** |  |  |  |  |  |  |  |  |
| Birth weight for gestational age (percentiles) |  |  |  |  |  |  |  |  |
| <3^rd^ | 80 924/3 | 1.33 (0.42-4.22) | 80 241/13 | 0.97 (0.56-1.68) | 73 177/11 | 0.96 (0.53-1.75) | 65 413/23 | 1.70 (1.12-2.59) |
| 3^rd^ to <10^th^ | 216 037/5 | 0.88 (0.35-2.17) | 214 774/22 | 0.64 (0.42-0.99) | 192 066/28 | 0.98 (0.67-1.44) | 167 594/31 | 0.92 (0.64-1.32) |
| ≥10^th^ | 3 498 642/86 | 1.0 | 3 484 292/499 | 1.0 | 3 053 878/409 | 1.0 | 2 578 060/480 | 1.0 |
| **Neurologic disease** |  |  |  |  |  |  |  |  |
| Birth weight for gestational age (percentiles) |  |  |  |  |  |  |  |  |
| <3^rd^ | 80 924/16 | 2.88 (1.73-4.81) | 80 241/7 | 1.69 (0.79-3.61) | 73 177/7 | 3.59 (1.64-7.87) | 65 413/7 | 1.38 (0.65-2.95) |
| 3^rd^ to <10^th^ | 216 037/27 | 1.87 (1.25-2.80) | 214 774/15 | 1.41 (0.83-2.40) | 192 066/9 | 1.84 (0.92-3.71) | 167 594/15 | 1.20 (0.71-2.04) |
| ≥10^th^ | 3 498 642/222 | 1.0 | 3 484 292/165 | 1.0 | 3 053 878/70 | 1.0 | 2 578 060/174 | 1.0 |

HR, hazard ratio; CI, confidence interval.

^*^ HRs were adjusted for maternal age, maternal education level (<10 years, 10-11 years, 12 years, 13-14 years, ≥15 years, or unknown), maternal country of birth (Nordic or non-Nordic country), maternal parity (1, 2-3, or ≥4), child’s sex, and calendar period of birth (1973-1976, every 5 years thereafter, or 2007-2012)
