# Supplementary material for: Small for gestational age and risk of childhood mortality: A Swedish population study
Source: PLoS Med. 2018 Dec 18;15(12):e1002717. doi: 10.1371/journal.pmed.1002717 (PMC6298647; doi:10.1371/journal.pmed.1002717)
Supplement: S3 Table — (DOCX) [file pmed.1002717.s008.docx]

**S3 Table. Association of small for gestational age (SGA) with the risk of childhood mortality by age groups, a cohort study of all live births without major malformations during 1973-2012 in Sweden. Analysis restricted to term births (≥37 gestational weeks).**

| **Age groups** | **Population analysis** | | | **Sibling analysis** | | |
| --- | --- | --- | --- | --- | --- | --- |
|  | **N of children** | **N of events** | **HR (95% CI)*^*^*** | **N of children**^†^ | **N of events**^†^ | **HR (95% CI)**^‡^ |
| **28 days to <18 years** |  |  |  |  |  |  |
| Birth weight for gestational age (percentiles) |  |  |  |  |  |  |
| <3^rd^ | 67 715 | 409 | 1.98 (1.79-2.19) | 457 | 235 | 1.85 (1.51-2.28) |
| 3^rd^ to <10^th^ | 203 076 | 734 | 1.29 (1.19-1.39) | 1 049 | 426 | 1.24 (1.08-1.42) |
| ≥10^th^ | 3 348 723 | 8 473 | 1.0 | 2 194 | 565 | 1.0 |
| **28 days to <1 year** |  |  |  |  |  |  |
| Birth weight for gestational age (percentiles) |  |  |  |  |  |  |
| <3^rd^ | 67 715 | 163 | 2.85 (2.43-3.34) | 209 | 97 | 1.98 (1.45-2.72) |
| 3^rd^ to <10^th^ | 203 076 | 273 | 1.67 (1.47-1.89) | 426 | 169 | 1.42 (1.14-1.76) |
| ≥10^th^ | 3 348 723 | 2 597 | 1.0 | 932 | 221 | 1.0 |
| **1 year to <5 years** |  |  |  |  |  |  |
| Birth weight for gestational age (percentiles) |  |  |  |  |  |  |
| <3^rd^ | 67 240 | 95 | 1.93 (1.57-2.37) | 102 | 59 | 2.24 (1.45-3.46) |
| 3^rd^ to <10^th^ | 201 941 | 164 | 1.20 (1.02-1.40) | 250 | 97 | 1.29 (0.96-1.72) |
| ≥10^th^ | 3 335 426 | 2 026 | 1.0 | 538 | 131 | 1.0 |
| **5 years to <10 years** |  |  |  |  |  |  |
| Birth weight for gestational age (percentiles) |  |  |  |  |  |  |
| <3^rd^ | 61 647 | 56 | 1.68 (1.28-2.20) | 47 | 28 | 2.03 (1.05-3.92) |
| 3^rd^ to <10^th^ | 180 742 | 115 | 1.28 (1.05-1.55) | 144 | 65 | 1.33 (0.92-1.93) |
| ≥10^th^ | 2 922 662 | 1 300 | 1.0 | 279 | 71 | 1.0 |
| **10 years to <18 years** |  |  |  |  |  |  |
| Birth weight for gestational age (percentiles) |  |  |  |  |  |  |
| <3^rd^ | 55 577 | 95 | 1.41 (1.15-1.73) | 105 | 51 | 1.31 (0.86-1.99) |
| 3^rd^ to <10^th^ | 157 978 | 182 | 1.01 (0.87-1.18) | 241 | 94 | 0.93 (0.70-1.24) |
| ≥10^th^ | 2 467 006 | 2 550 | 1.0 | 451 | 148 | 1.0 |

HR, hazard ratio; CI, confidence interval.

*^*^* HRs in the population analysis were adjusted for maternal age, maternal education level (<10 years, 10-11 years, 12 years, 13-14 years, ≥15 years, or unknown), maternal country of birth (Nordic or non-Nordic country), maternal parity (1, 2-3, or ≥4), child’s sex, and calendar period of birth (1973-1976, every 5 years thereafter, or 2007-2012).

^†^ In the within-sibling analysis, number of births represents the informative siblings, namely siblings who were discordant for both exposure (SGA vs. non-SGA) and outcome (death or alive) in order to contribute to the risk estimates, although all children with siblings were included for analysis.

^‡^ HRs in the sibling analyses were adjusted for maternal age and child’s sex.
