# Supplementary material for: Small for gestational age and risk of childhood mortality: A Swedish population study
Source: PLoS Med. 2018 Dec 18;15(12):e1002717. doi: 10.1371/journal.pmed.1002717 (PMC6298647; doi:10.1371/journal.pmed.1002717)
Supplement: S4 Table — (DOCX) [file pmed.1002717.s009.docx]

**S4 Table. Association of small for gestational age (SGA) with the risk of childhood mortality (age from 28 days to <18 years) by underlying cause of death, a cohort study of all live births without major malformations during 1973-2012 in Sweden. Analysis restricted to term births (≥37 gestational weeks).**

| **Cause of death** | **Population analysis** | | | **Sibling analysis** | | |
| --- | --- | --- | --- | --- | --- | --- |
|  | **N of children** | **N of events** | **HR (95% CI)*^*^*** | **N of children**^†^ | **N of events**^†^ | **HR (95% CI)**^†^ |
| **Infection** |  |  |  |  |  |  |
| Birth weight for gestational age (percentiles) |  |  |  |  |  |  |
| <3^rd^ | 67 715 | 40 | 2.20 (1.60-3.03) | 38 | 22 | 3.96 (1.78-8.82) |
| 3^rd^ to <10^th^ | 203 076 | 73 | 1.45 (1.14-1.85) | 113 | 43 | 1.19 (0.77-1.83) |
| ≥10^th^ | 3 348 723 | 728 | 1.0 | 250 | 57 | 1.0 |
| **Injury** |  |  |  |  |  |  |
| Birth weight for gestational age (percentiles) |  |  |  |  |  |  |
| <3^rd^ | 67 715 | 98 | 1.32 (1.08-1.62) | 97 | 46 | 1.21 (0.78-1.86) |
| 3^rd^ to <10^th^ | 203 076 | 211 | 1.08 (0.94-1.25) | 288 | 109 | 1.01 (0.78-1.32) |
| ≥10^th^ | 3 348 723 | 2 720 | 1.0 | 545 | 158 | 1.0 |
| **Cancer** |  |  |  |  |  |  |
| Birth weight for gestational age (percentiles) |  |  |  |  |  |  |
| <3^rd^ | 67 715 | 43 | 1.23 (0.90-1.66) | 53 | 26 | 1.52 (0.81-2.85) |
| 3^rd^ to <10^th^ | 203 076 | 84 | 0.87 (0.70-1.08) | 125 | 53 | 0.98 (0.65-1.46) |
| ≥10^th^ | 3 348 723 | 1 407 | 1.0 | 245 | 77 | 1.0 |
| **Neurologic disease** |  |  |  |  |  |  |
| Birth weight for gestational age (percentiles) |  |  |  |  |  |  |
| <3^rd^ | 67 715 | 28 | 2.06 (1.41-3.02) | 25 | 15 | 2.32 (0.92-5.85) |
| 3^rd^ to <10^th^ | 203 076 | 51 | 1.34 (1.01-1.79) | 66 | 30 | 1.41 (0.82-2.42) |
| ≥10^th^ | 3 348 723 | 567 | 1.0 | 134 | 34 | 1.0 |

HR, hazard ratio; CI, confidence interval.

*^*^* HRs in the population analysis were adjusted for maternal age, maternal education level (<10 years, 10-11 years, 12 years, 13-14 years, ≥15 years, or unknown), maternal country of birth (Nordic or non-Nordic country), maternal parity (1, 2-3, or ≥4), child’s sex, and calendar period of birth (1973-1976, every 5 years thereafter, or 2007-2012).

^†^ In the within-sibling analysis, number of births represents the informative siblings, namely siblings who were discordant for both exposure (SGA vs. non-SGA) and outcome (death or alive) in order to contribute to the risk estimates, although all children with siblings were included for analysis.

^‡^ HRs in the sibling analyses were adjusted for maternal age and child’s sex.
