# Supplementary material for: Small for gestational age and risk of childhood mortality: A Swedish population study
Source: PLoS Med. 2018 Dec 18;15(12):e1002717. doi: 10.1371/journal.pmed.1002717 (PMC6298647; doi:10.1371/journal.pmed.1002717)
Supplement: S5 Table — (DOCX) [file pmed.1002717.s010.docx]

**S5 Table.** **Association of small for gestational age (SGA) with the risk of childhood mortality (age from 28 days to <18 years) by underlying cause of death and modality of delivery, a cohort study of all live births without major malformations during 1973-2012 in Sweden.**

| **Cause of death** | **Population analysis** | | | **Sibling analysis** | | |
| --- | --- | --- | --- | --- | --- | --- |
|  | **N of children** | **N of events** | **HR (95% CI)*^*^*** | **N of children**^†^ | **N of events**^†^ | **HR (95% CI)**^†^ |
| **All causes** |  |  |  |  |  |  |
| ***Vaginal delivery*** |  |  |  |  |  |  |
| Birth weight for gestational age (percentiles) |  |  |  |  |  |  |
| <3^rd^ | 56 980 | 365 | 2.00 (1.80-2.23) | 392 | 193 | 1.74 (1.40-2.17) |
| 3^rd^ to <10^th^ | 185 086 | 706 | 1.31 (1.21-1.42) | 1 014 | 409 | 1.25 (1.09-1.44) |
| ≥10^th^ | 3 094 500 | 8 173 | 1.0 | 2 056 | 537 | 1.0 |
| ***Caecarean section*** |  |  |  |  |  |  |
| Birth weight for gestational age (percentiles) |  |  |  |  |  |  |
| <3^rd^ | 23 944 | 277 | 3.55 (3.11-4.05) | 120 | 90 | 4.75 (2.95-7.67) |
| 3^rd^ to <10^th^ | 30 951 | 157 | 1.61 (1.36-1.91) | 87 | 39 | 1.83 (1.11-3.01) |
| ≥10^th^ | 404 142 | 1 160 | 1.0 | 229 | 39 | 1.0 |
|  |  |  |  |  |  |  |
| **Infection** |  |  |  |  |  |  |
| ***Vaginal delivery*** |  |  |  |  |  |  |
| Birth weight for gestational age (percentiles) |  |  |  |  |  |  |
| <3^rd^ | 56 980 | 43 | 2.59 (1.90-3.53) | 37 | 22 | 4.04 (1.79-9.13) |
| 3^rd^ to <10^th^ | 185 086 | 74 | 1.51 (1.19-1.92) | 120 | 43 | 1.13 (0.74-1.73) |
| ≥10^th^ | 3 094 500 | 721 | 1.0 | 255 | 60 | 1.0 |
| ***Caecarean section*** |  |  |  |  |  |  |
| Birth weight for gestational age (percentiles) |  |  |  |  |  |  |
| <3^rd^ | 23 944 | 31 | 3.43 (2.31-5.11) | 15 | 10 | 4.06 (1.16-14.25) |
| 3^rd^ to <10^th^ | 30 951 | 22 | 1.95 (1.23-3.07) | 15 | 6 | 1.23 (0.36-4.26) |
| ≥10^th^ | 404 142 | 130 | 1.0 | 41 | 8 | 1.0 |
|  |  |  |  |  |  |  |
| **Injury** |  |  |  |  |  |  |
| ***Vaginal delivery*** |  |  |  |  |  |  |
| Birth weight for gestational age (percentiles) |  |  |  |  |  |  |
| <3^rd^ | 56 980 | 88 | 1.35 (1.09-1.67) | 84 | 42 | 1.34 (0.85-2.12) |
| 3^rd^ to <10^th^ | 185 086 | 196 | 1.07 (0.93-1.24) | 273 | 99 | 0.94 (0.72-1.23) |
| ≥10^th^ | 3 094 500 | 2 612 | 1.0 | 499 | 149 | 1.0 |
| ***Caecarean section*** |  |  |  |  |  |  |
| Birth weight for gestational age (percentiles) |  |  |  |  |  |  |
| <3^rd^ | 23 944 | 25 | 1.25 (0.83-1.89) | 7 | 3 | 1.47 (0.24-9.02) |
| 3^rd^ to <10^th^ | 30 951 | 27 | 1.10 (0.74-1.64) | 8 | 6 | 10.11 (1.12-91.08) |
| ≥10^th^ | 404 142 | 259 | 1.0 | 16 | 4 | 1.0 |
|  |  |  |  |  |  |  |
| **Cancer** |  |  |  |  |  |  |
| ***Vaginal delivery*** |  |  |  |  |  |  |
| Birth weight for gestational age (percentiles) |  |  |  |  |  |  |
| <3^rd^ | 56 980 | 39 | 1.30 (0.94-1.79) | 47 | 25 | 2.03 (1.04-3.98) |
| 3^rd^ to <10^th^ | 185 086 | 75 | 0.85 (0.67-1.07) | 107 | 44 | 1.02 (0.66-1.59) |
| ≥10^th^ | 3 094 500 | 1 320 | 1.0 | 214 | 63 | 1.0 |
| ***Caecarean section*** |  |  |  |  |  |  |
| Birth weight for gestational age (percentiles) |  |  |  |  |  |  |
| <3^rd^ | 23 944 | 11 | 0.99 (0.54-1.83) | 7 | 3 | 0.87 (0.14-5.59) |
| 3^rd^ to <10^th^ | 30 951 | 11 | 0.80 (0.43-1.47) | 11 | 6 | 1.08 (0.30-3.94) |
| ≥10^th^ | 404 142 | 154 | 1.0 | 18 | 6 | 1.0 |
|  |  |  |  |  |  |  |
| **Neurologic disease** |  |  |  |  |  |  |
| ***Vaginal delivery*** |  |  |  |  |  |  |
| Birth weight for gestational age (percentiles) |  |  |  |  |  |  |
| <3^rd^ | 56 980 | 24 | 2.01 (1.33-3.04) | 26 | 12 | 1.50 (0.62-3.60) |
| 3^rd^ to <10^th^ | 185 086 | 52 | 1.45 (1.09-1.93) | 68 | 32 | 1.59 (0.92-2.76) |
| ≥10^th^ | 3 094 500 | 544 | 1.0 | 124 | 32 | 1.0 |
| ***Caecarean section*** |  |  |  |  |  |  |
| Birth weight for gestational age (percentiles) |  |  |  |  |  |  |
| <3^rd^ | 23 944 | 13 | 2.36 (1.31-4.25) | 9 | 7 | 4.31 (0.69-26.88) |
| 3^rd^ to <10^th^ | 30 951 | 14 | 2.01 (1.14-3.56) | 8 | 4 | 2.31 (0.43-12.46) |
| ≥10^th^ | 404 142 | 87 | 1.0 | 16 | 3 | 1.0 |

HR, hazard ratio; CI, confidence interval.

*^*^* HRs in the population analysis were adjusted for maternal age, maternal education level (<10 years, 10-11 years, 12 years, 13-14 years, ≥15 years, or unknown), maternal country of birth (Nordic or non-Nordic country), maternal parity (1, 2-3, or ≥4), child’s sex, and calendar period of birth (1973-1976, every 5 years thereafter, or 2007-2012).

^†^ In the within-sibling analysis, number of births represents the informative siblings, namely siblings who were discordant for both exposure (SGA vs. non-SGA) and outcome (death or alive) in order to contribute to the risk estimates, although all children with siblings were included for analysis.

^‡^ HRs in the sibling analyses were adjusted for maternal age and child’s sex.
