# Supplementary material for: Small for gestational age and risk of childhood mortality: A Swedish population study
Source: PLoS Med. 2018 Dec 18;15(12):e1002717. doi: 10.1371/journal.pmed.1002717 (PMC6298647; doi:10.1371/journal.pmed.1002717)
Supplement: S6 Table — (DOCX) [file pmed.1002717.s011.docx]

**S6 Table. Association of small for gestational age (SGA) with the risk of childhood mortality (age from 28 days to <18 years) by age at follow-up, by calendar periods of childbirth, a cohort study of all live births without major malformations during 1973-2012 in Sweden.**

|  | **28 days to <1 year** | | **1 year to <5 years** | | **5 years to <10 years** | | **10 years to <18 years** | |
| --- | --- | --- | --- | --- | --- | --- | --- | --- |
| **Calendar periods of childbirth** | **N children /events** | **HR (95% CI)*^*^*** | **N children /events** | **HR (95% CI)*^*^*** | **N children /events** | **HR (95% CI)*^*^*** | **N children /events** | **HR (95% CI)*^*^*** |
| **Born from 1973 to 1981** |  |  |  |  |  |  |  |  |
| Birth weight for gestational age (percentiles) |  |  |  |  |  |  |  |  |
| <3^rd^ | 28 364/116 | 3.66 (3.01-4.45) | 28 117/55 | 1.94 (1.48-2.56) | 27 686/38 | 1.92 (1.38-2.68) | 27 334/60 | 1.41 (1.08-1.83) |
| 3^rd^ to <10^th^ | 66 310/125 | 1.68 (1.39-2.03) | 65 811/76 | 1.17 (0.92-1.48) | 64 713/50 | 1.11 (0.83-1.49) | 63 905/99 | 1.02 (0.83-1.25) |
| ≥10^th^ | 743 452/846 | 1.0 | 739 182/723 | 1.0 | 727 752/500 | 1.0 | 719 059/1 073 | 1.0 |
| **Born from 1982 to 1991** |  |  |  |  |  |  |  |  |
| Birth weight for gestational age (percentiles) |  |  |  |  |  |  |  |  |
| <3^rd^ | 21 726/100 | 3.65 (2.97-4.48) | 21 545/37 | 2.42 (1.73-3.38) | 21 232/18 | 1.64 (1.02-2.63) | 20 972/36 | 1.44 (1.03-2.01) |
| 3^rd^ to <10^th^ | 56 356/114 | 1.57 (1.30-1.91) | 56 030/58 | 1.47 (1.12-1.92) | 55 180/39 | 1.40 (1.01-1.94) | 54 496/67 | 1.04 (0.81-1.34) |
| ≥10^th^ | 893 281/1 216 | 1.0 | 889 188/620 | 1.0 | 878 066/434 | 1.0 | 868 214/1 027 | 1.0 |
| **Born from 1992 to 2001** |  |  |  |  |  |  |  |  |
| Birth weight for gestational age (percentiles) |  |  |  |  |  |  |  |  |
| <3^rd^ | 15 109/69 | 7.00 (5.44-9.00) | 14 959/14 | 1.76 (1.03-3.00) | 14 671/3 | 0.50 (0.16-1.58) | 14 495/20 | 2.03 (1.30-3.18) |
| 3^rd^ to <10^th^ | 42 918/64 | 2.21 (1.71-2.87) | 42 670/29 | 1.27 (0.87-1.86) | 41 900/18 | 1.07 (0.67-1.73) | 41 342/25 | 0.88 (0.59-1.32) |
| ≥10^th^ | 851 899/596 | 1.0 | 848 390/440 | 1.0 | 835 582/320 | 1.0 | 826 566/583 | 1.0 |
| **Born from 2002 to 2012** |  |  |  |  |  |  |  |  |
| Birth weight for gestational age (percentiles) |  |  |  |  |  |  |  |  |
| <3^rd^ | 15 725/51 | 7.43 (5.54-9.96) | 15 620/20 | 3.16 (2.01-4.97) | 9 588/5 | 2.55 (1.04-6.27) | 2 612/0 | - |
| 3^rd^ to <10^th^ | 50 453/62 | 2.82 (2.16-3.69) | 50 263/25 | 1.25 (0.83-1.87) | 30 273/12 | 1.99 (1.09-3.63) | 7 851/0 | - |
| ≥10^th^ | 1 010 010/446 | 1.0 | 1 007 532/385 | 1.0 | 612 478/114 | 1.0 | 164 221/10 | 1.0 |

HR, hazard ratio; CI, confidence interval.

*^*^* HRs were adjusted for maternal age, maternal education level (<10 years, 10-11 years, 12 years, 13-14 years, ≥15 years, or unknown), maternal country of birth (Nordic or non-Nordic country), maternal parity (1, 2-3, or ≥4), child’s sex, and calendar period of birth (1973-1976, every 5 years thereafter, or 2007-2012).
