# Supplementary material for: Small for gestational age and risk of childhood mortality: A Swedish population study
Source: PLoS Med. 2018 Dec 18;15(12):e1002717. doi: 10.1371/journal.pmed.1002717 (PMC6298647; doi:10.1371/journal.pmed.1002717)
Supplement: S7 Table — (DOC) [file pmed.1002717.s012.doc]

**S7 Table. Association of small for gestational age (SGA) with the risk of childhood mortality by age groups, a cohort study of all live births without major malformations during 1992-2012 in Sweden. Analyses were further adjusted for maternal smoking and body mass index (BMI). Births with missing maternal BMI (n=255 589, 12.9%) were excluded in the analysis.**

|  | **N of children** | **N of events** | **HR (95% CI)*^*^*** | **HR (95% CI)** ^†^ | **HR (95% CI)**^‡^ |
| --- | --- | --- | --- | --- | --- |
| **Population analysis** |  |  |  |  |  |
| Birth weight for gestational age (percentiles) |  |  |  |  |  |
| <3^rd^ | 26,396 | 139 | 3.47 (2.92-4.12) | 3.22 (2.71-3.83) | 3.24 (2.73-3.85) |
| 3^rd^ to <10^th^ | 80,927 | 193 | 1.59 (1.38-1.85) | 1.50 (1.30-1.74) | 1.52 (1.31-1.76) |
| ≥10^th^ | 1,623,202 | 2,431 | 1.0 | 1.0 | 1.0 |
|  |  |  |  |  |  |
| **Sibling analysis** |  |  |  |  |  |
| Birth weight for gestational age (percentiles) |  |  |  |  |  |
| <3^rd^ | 105 | 76 | 5.14 (3.06-8.66) | 5.24 (3.11-8.83) | 5.19 (3.07-8.76) |
| 3^rd^ to <10^th^ | 206 | 104 | 1.97 (1.43-2.73) | 1.97 (1.43-2.73) | 1.94 (1.40-2.69) |
| ≥10^th^ | 405 | 82 | 1.0 | 1.0 | 1.0 |

HR, hazard ratio; CI, confidence interval.

*^*^* HRs in the population analysis were adjusted for maternal age, maternal education level (<10 years, 10-11 years, 12 years, 13-14 years, ≥15 years, or unknown), maternal country of birth (Nordic or non-Nordic country), maternal parity (1, 2-3, or ≥4), child’s sex, and calendar period of birth (1973-1976, every 5 years thereafter, or 2007-2012). HRs in the sibling analyses were adjusted for maternal age and child’s sex.

^†^ HRs in both population and sibling analyses were additionally adjusted for smoking during pregnancy (no smoking, smoking, or unknown; 1.2% missing during 1992-2012).

^‡^ HRs in both population and sibling analyses were additionally adjusted for smoking during pregnancy (no smoking, smoking, or unknown) and BMI at early pregnancy. We applied restricted cubic splines on BMI with four knots placed at 0.05, 0.35, 0.65 and 0.95 quantiles of the distribution of outcome events. BMI was calculated from the information on height and weight recorded at the first prenatal visit from 1992 (12.9% missing during 1992-2012).
